# EOR-1 and EOR-2 function in RMED/V neuron specification

**DOI:** 10.17912/micropub.biology.000138

**Published:** 2019-07-31

**Authors:** Xun Huang, Yishi Jin

**Affiliations:** 1 MCD biology, University of California, Santa Cruz, CA95064; 2 Institute of Genetics and Developmental Biology, Chinese Academy of Sciences, Beijing, 100101, China; 3 Neurobiology Section, Division of Biological Sciences, University of California, San Diego, CA92093

**Figure 1:**
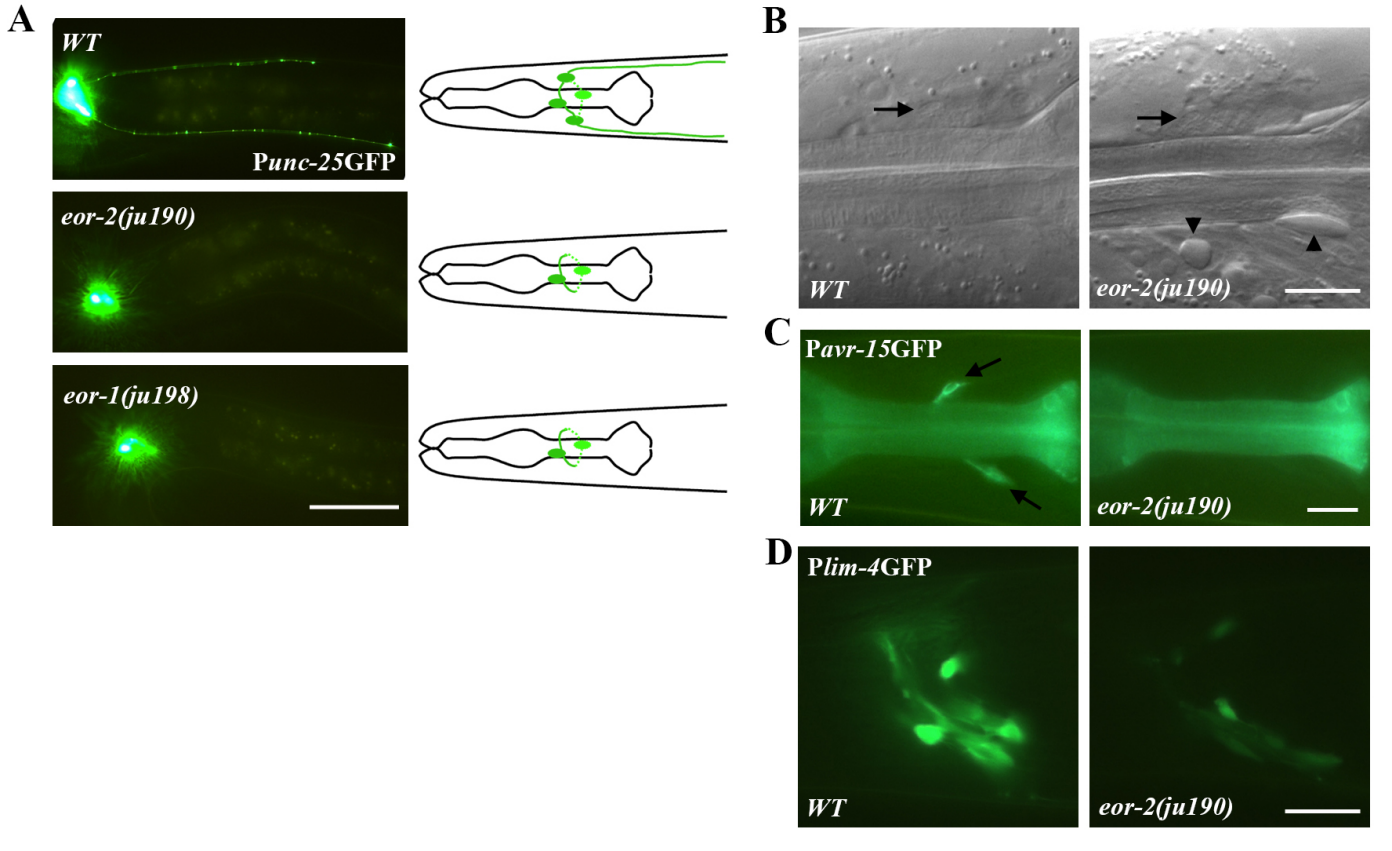
Mutants affecting RMED/V neuron specification. (A) P*_unc-25_*GFP expression in different mutants. Schematic illustrations of RME cell morphology in wild type (*WT*) and mutants are in the right. Scale bar: 50µm. (B) RMED cells (Arrowed) in wild type and *eor-2(ju190)* animals. Arrowheads point to abnormal large vesicles accumulating in the head of *eor-2(ju190)* animals. Scale bar: 10µm. (C) Expression of P*_avr-15_*GFP in wild type and *eor-2(ju190)* animals. P*_avr-15_*GFP is expressed in RMED/V neurons in wild type, while the expression is lost in *eor-2(ju190)* animals. Scale bar: 10µm. (D) Expression of P*_lim-4_*GFP in wild type and *eor-2(ju190)* animals. P*_lim-4_*GFP is brightly expressed in some neurons in the head region in wild type, while the expression is attenuated in *eor-2(ju190)* animals. Scale bar: 10µm. (A-D) All the images were taken at young adult stage.

## Description

In a visual screen for genes that regulate the pattern of the *juIs76*[P*_unc-25_*GFP] marker, which labels four GABAergic RME neurons and 19 ventral cord D-type neurons (Huang *et al.*, 2002), we isolated two mutants, *eor-2(ju190)* and *eor-1(ju198)* (Huang and Jin, 2019). In both *eor-2(ju190)* and *eor-1(ju198)* mutants, P*_unc-25_*GFP expression was almost completely abolished in RMED/V cells, whereas RMEL/R cells and the D neurons showed normal morphology (Figure 1A). We observed similar defects with a different P*_unc-25_*GFP transgene. The absence of P*_unc-25_*GFP expression was seen in all larval stages and adults, was more frequent in RMED than in RMEV cells. For example, 98% of *eor-1(ju198)* animals lost P*_unc-25_*GFP expression in RMED and 67% in RMEV (N=100). *ju198* behaves as a partial loss of function mutation because 100% and 94% of *eor-1(cs28)* animals do not express P*_unc-25_*GFP expression in RMED and RMEV, respectively (N=100) (Huang and Jin, 2019). *eor-2(ju190)*animals also displayed mild Unc, low penetrant Egl and rod-like lethality. The loss of P*_unc-25_*GFP expression in *eor-2(ju190)* and *eor-1(ju198)* could be due to cell fate alterations or cell death. To distinguish between these possibilities, we first examined the cell body positions of RMED and RMEV cells under Nomarski microscope (Huang *et al.*, 2004). In both *eor-2(ju190)* and *eor-1(ju198)* mutants, the RMED and RMEV cells were found in their normal locations (Figure 1B). We also made double mutants of *eor-2(ju190)* and *ced-3(n717)*, which blocks apoptosis, and found that *eor-2(ju190); ced-3(n717)* double mutants showed absence of P*_unc-25_*GFP expression in RMED/V, similar to *eor-2(ju190)* single mutants, indicating that in*eor-2(ju190)* and *eor-1(ju198)* animals, the RMED and RMEV cells are alive, but that their differentiated traits are likely altered.

To further examine whether other properties of the RMED/V cells might be altered in these mutants, we looked at the expression of P*_avr-15_*GFP, which is normally expressed in both RMED and RMEV neurons (Dent *et al.*, 1997) and P*_lim-4_*GFP transgenes, which is normally expressed in RMEV neuron and some other head neurons (Sagasti *et al.*, 1999). We found that in *eor-2(ju190)* animals,P*_avr-15_*GFP was not expressed in RMED/V (Figure 1C), the GFP intensity from P*_lim-4_*GFP transgene was greatly reduced, but not abolished, in all expressing cells (Figure 1D). These data show that *eor-2(ju190)* alters multiple differentiated aspects of RMED/V neurons.

## Description

Strains are: CZ2014 *eor-1(ju198), juIs76*; CZ2006 *eor-2(ju190); juIs76*.

The GFP reporters used are listed below: *juIs76[P_unc-25_GFP]*; *juIs73[P_unc-25_GFP]*; *P_avr-15_GFP*; *P_lim-4_GFP.*
